# Rapid Evolution of the Sequences and Gene Repertoires of Secreted Proteins in Bacteria

**DOI:** 10.1371/journal.pone.0049403

**Published:** 2012-11-26

**Authors:** Teresa Nogueira, Marie Touchon, Eduardo P. C. Rocha

**Affiliations:** 1 Centro de Biologia Ambiental, Evolutionary Ecology of Microorganisms, Faculdade de Ciências da Universidade de Lisboa, Lisboa, Portugal; 2 Instituto Gulbenkian de Ciência, Oeiras, Portugal; 3 Centro de Investigação em Saúde e Ambiente, Escola Superior de Tecnologia da Saúde do Porto, Instituto Politécnico do Porto, Vila Nova de Gaia, Portugal; 4 Institut Pasteur, Microbial Evolutionary Genomics, Département Génomes et Génétique, Paris, France; 5 CNRS, UMR3525, Paris, France; University Of Montana - Missoula, United States of America

## Abstract

Proteins secreted to the extracellular environment or to the periphery of the cell envelope, the secretome, play essential roles in foraging, antagonistic and mutualistic interactions. We hypothesize that arms races, genetic conflicts and varying selective pressures should lead to the rapid change of sequences and gene repertoires of the secretome. The analysis of 42 bacterial pan-genomes shows that secreted, and especially extracellular proteins, are predominantly encoded in the accessory genome, i.e. among genes not ubiquitous within the clade. Genes encoding outer membrane proteins might engage more frequently in intra-chromosomal gene conversion because they are more often in multi-genic families. The gene sequences encoding the secretome evolve faster than the rest of the genome and in particular at non-synonymous positions. Cell wall proteins in Firmicutes evolve particularly fast when compared with outer membrane proteins of Proteobacteria. Virulence factors are over-represented in the secretome, notably in outer membrane proteins, but cell localization explains more of the variance in substitution rates and gene repertoires than sequence homology to known virulence factors. Accordingly, the repertoires and sequences of the genes encoding the secretome change fast in the clades of obligatory and facultative pathogens and also in the clades of mutualists and free-living bacteria. Our study shows that cell localization shapes genome evolution. In agreement with our hypothesis, the repertoires and the sequences of genes encoding secreted proteins evolve fast. The particularly rapid change of extracellular proteins suggests that these public goods are key players in bacterial adaptation.

## Introduction

Prokaryotes secrete effector molecules to the environment and to exposed regions in the cell envelope to change their niche, scavenge resources and to interact with other organisms. Some of such functions require the secretion of proteins across the cell envelope either to the periphery of the cell, the cell wall in monoderms and the outer membrane in diderms, or to the extracellular environment. Secreted proteins perform a variety of important functions. They provide antibiotic resistance [1], protect against protozoa [2], antagonize bacterial competitors [3], and mediate mutualistic associations [4]. Importantly, many secreted proteins have been described as virulence factors allowing pathogens to evade immune responses and exploit or kill eukaryotic cells [5], [6]. Indeed, most past work in protein secretion was motivated by the key role of secreted proteins (the secretome) in pathogenesis.

The very large size of typical bacterial populations compensates the reduced impact of a single bacterial cell on its environment. Thus, most of the environmentally relevant bacterial processes are social [7]–[9]. This is particularly true for processes involving secreted proteins, and especially extracellular proteins, because they are costly public goods. Protein secretion is costly because of the complexity of secretion systems, the energy required to translocate effectors and because secreted proteins are lost for the cell. For example, in *Salmonella enterica* Typhymurium the expression of the type 3 secretion system 1 (T3SS-1) was found to double the generation time [10]. The production of costly public goods poses social dilemmas because bacteria not participating in secretion of the public good outcompete the populations of producers (cooperative bacteria) by reaping the benefits of cooperation without paying its costs [11]. The disruption of these social processes may lead to population extinction (tragedy of the commons) [12], [13].

Horizontal transfer of social traits favors the emergence and the stabilization of cooperative behaviors [14]. First, transfer of a social trait by mobile genetic elements increases the frequency of the trait in the population (infectiousness) [11]. Second, social traits shared in the community by recent transfer show high genetic relatedness and are thus favored by kin selection [15]. In both cases, the theoretical prediction is that high transfer rates of social traits promote cooperative behavior. Hence, we expect genes involved in social interactions, e.g. exposed proteins, and especially genes encoding public goods, i.e. extracellular proteins, to be transferred at high rates. This is indeed observed in *Escherichia coli*, where the density of genes encoding secreted proteins is strongly related to the genetic mobility of the loci, i.e. the highest density of genes encoding secreted proteins is found in the regions of the genome that are gained and lost at higher rates [14]. Genes encoding secreted proteins might also be frequently lost for two reasons. First, mobile elements are often lost. Second, intra- and inter-genomic genetic conflicts are particularly important for social traits [16]. Such conflicts might precipitate their loss. Hence, within all gene families in a given clade - its pan-genome [17] - we expect genes encoding secreted proteins to be more frequent in the accessory genome (genes present in a subset of strains) than in the core genome (genes ubiquitous in the clade). Many examples support this expectation: (i) antibiotic resistance via secretion of β-lactamases is typically spread by mobile elements [18]; (ii) colicins are generally encoded in plasmids [19]; (iii) genes encoding secreted proteins are over-represented in super-integrons [20].

Bacteria are constantly engaging in evolutionary arms races with their parasites, their hosts and their predators [21], [22]. Many of these ecological interactions involve secreted proteins. Hence, secreted proteins are expected to be under particularly strong diversifying and/or positive selection. Rapid evolution caused by direct cell-to-cell interactions should affect especially the proteins exposed at the cell surface [23]–[26]. Accordingly, many secreted proteins are found in mobile genetic elements such as prophages or genomic islands [27], [28]. Hypermutable regions that allow rapid change of gene expression patterns are frequent among genes encoding cell envelope proteins [29], [30]. A study aiming at identifying bacterial proteins under diversifying selection showed that 5 out of 7 cases in Chlamydiacea and 7 of the 11 cases in *Pyrococcus* concerned membrane or secreted proteins [31]. Similarly, another study showed rapid substitution rates in outer membranes of Chlamydiacea [32]. In *E. coli* and *B. subtilis*, cell envelope proteins were found to evolve faster than the average protein after accounting for essentiality and expression levels [33]. In *Pseudomonas aeruginosa* extracellular and outer membrane proteins evolve faster than cytoplasmic proteins [34]. A scan in *Photobacterium profundum* SS9 and *Shewanella benthica* KT99 showed more frequent positive selection in genes encoding functions related to motility and transport [35], thus including many cell envelope-associated proteins. Finally, recombination leading to genetic diversification was found predominantly in genes encoding cell envelope proteins in *Mycobacterium tuberculosis* [36] and key antigens in *Streptococcus pneumoniae* [37]. In mammals, secreted proteins evolve faster and their substitution rates are correlated with tissue specificity, even when controlling for expression levels and protein-protein-interaction data [38], [39].

The previous examples suggest that protein localization shapes the rate of change of gene repertoires and sequences in bacteria. Yet, the pervasiveness of this effect has not been tested. In this work we do a systematic analysis of genetic diversification of proteins in function of their cell localization in both monoderms and diderms. Our dataset includes a large fraction of the most significant bacterial human pathogens and therefore we analyze the diversification of virulence factors in the light of their cellular localization. Thanks to this, we can explicitly link gene repertoires, sequence plasticity and the evolution of virulence factors in bacterial pathogens. Our work is aimed at testing the hypothesis that both key evolutionary processes among prokaryotes, accumulation of substitutions and horizontal gene transfer, drive rapid evolution of the secretome.

## Results

### Localization of the Proteins Encoded by the Pan-genome

We retrieved from GenBank all completely sequenced chromosomes and plasmids of Firmicutes (monoderms) and Proteobacteria (diderms). They include most of the best-studied prokaryotes in terms of secretion and virulence. We put together the gene repertoires of chromosomes (but excluding the 2.6% of genes encoded in plasmids, see below) of closely related taxa within these phyla to compute their pan-genomes. A widely accepted concept of species for prokaryotes is lacking [40], [41]. Hence, we put together the genomes with 16S rRNA sequence identity higher than 98.7%. This is a good compromise with present species definitions in bacteria [42] (see Methods). This definition allowed grouping together genomes that have different named species, e.g. *Bacillus cereus* and *B. anthracis*, but that are generally considered a single species. In order to accommodate the imprecision associated with measures of divergence based on a single gene (the 16S), the sequence similarity threshold was lowered to 98% if and only if the compared genomes had the same species name [43]. Inversely, when the core genome of a clade produced a phylogenetic tree with very long branches for some genomes (genetic distances >0.1) these were excluded. For example, the phylogenetic tree produced with the core genome of the *Salmonella* spp. presented *S. bongori* with a large terminal branch (>0.1 subst/nt) and the genome was thus excluded even if it respected the 16S rRNA similarity criterion. To control for the effect of strain choice, we re-did all major analyses in this work putting together in clades only the genomes with the same species name. We found qualitatively similar results (data not shown). We thus defined 42 groups (clades): 28 of Proteobacteria and 14 of Firmicutes. These clades contain between 4 and 47 genomes (Tables 1, S1 and S2), for a total of 421 genomes (36% of the available genomes). The pan-genomes include 231,096 protein families (see Methods, Table S3), of which 37% are in the core genomes and 63% in the accessory genomes. The fraction of the accessory genes in the 42 pan-genomes is highly variable from 85% (*E. coli*) to below 40% (e.g. *Listeria*) (details in Tables S1 and S2).

**Table 1:** Dataset statistics.

Phylum	Clades (genomes)	Localized Proteins	Pangenome (no. genes)^a^		Multigene families a,b	Homologs of VF a
			Core a	Accessory^a^		
Proteobacteria	28 (289)	100,613	47,765	52,848	5,752 (6%)	15,856
Firmicutes	14 (141)	45,687	20,962	24,725	2,535 (5%)	5,158
Total	42 (421)	146,300	68,727	77,573	8,287 (6%)	21,014

See Tables S1, S2 and S3 for more details.

a restricted to proteins with prediction of localization; ^b^ (%) of the localized proteins.

To study the evolutionary patterns of the secretome we identified the cell localization of the proteins encoded by the pan-genomes with PsortB [44], [45]. This software uses several complementary approaches to achieve ∼98% accuracy for positive predictions. Unreliably classed proteins, i.e. negative predictions, were removed from the analysis leaving a dataset of 146,300 families, i.e. 63% of the total. Non-localized proteins included a large majority of unknown function genes. Around 81% of non-localized proteins were encoded in the accessory genome. Accordingly, the fraction of proteins for which we could predict protein cell localization was 2.6 times higher for the core than for the accessory genomes (p<0.0001 for every clade, χ^2^ tests on contingency tables after Bonferroni correction). The number of localized proteins per clade varied proportionally with the clade’s average genome size (R^2^ = 0.77, p<0.0001). The slope of the regression of genome size and the number of identified proteins size was not significantly different from 1 (slope = 1.104, p>0.05, t-student). Hence, the fraction of genes that cannot be classed is higher in the accessory genome but is not affected by genome size.

**Figure 1:**
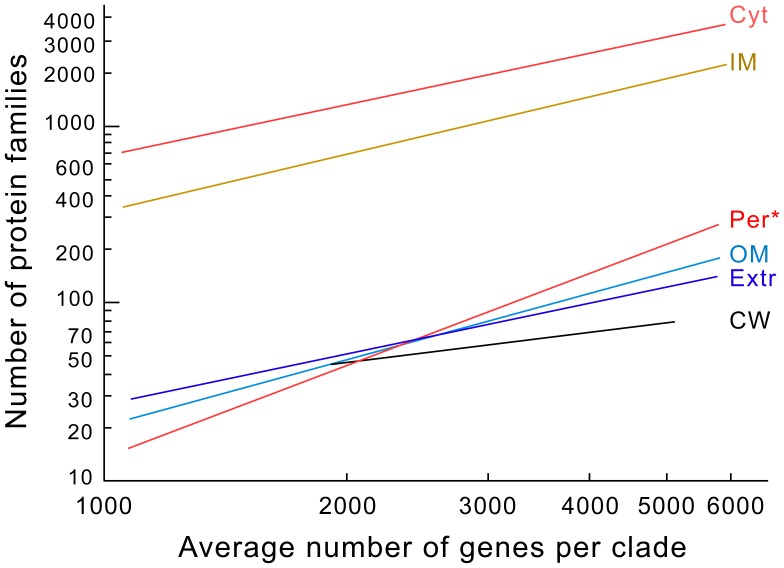
Linear regressions of the number of protein families per cell location in function of the average number of genes in a clade. Data points were removed for clarity. The absolute, but not the relative, frequency of proteins in each localization increases with the number of genes in genomes. Only the percentage of periplasmic proteins (indicated with an *) shows a significant correlation with genome size (Spearman’s rho p<0.001, after Bonferroni correction for multiple tests). The slope of the increase in the number of proteins with a given localization was not significantly different from the average trend for the other cell localizations (p>0.05, same test). Abbreviations of cell localizations: cytoplasm (Cyt), inner membrane (IM), periplasm (Per, Proteobacteria), cell wall (CW, Firmicutes), outer membrane (OM, Proteobacteria) and extracellular (Extr).

The results of PsortB suggest that most proteins are localized in the cytoplasm or in the inner membrane (resp. 60 and 33%). In Firmicutes, cell wall and extracellular proteins account resp. for 1.8% and 3.3% of all localized proteins, whereas in Proteobacteria the outer membrane and extracellular proteins account for resp. 2.5% and 1.8%. Genome size varies widely between and within the clades of our dataset. Larger genomes have higher rates of horizontal gene transfer [46]. Hence, if larger genomes have disproportionally more secreted proteins the over-representation of the secretome in the accessory genome could result trivially from the effect of genome size. We find that the fraction of the genome encoding the secretome is not significantly correlated with genome size (Spearman rho = 0.076, p = 0.63). This is true also for all the three locations, i.e. extracellular, cell wall and outer membrane, when taken separately (all p>0.05 after Bonferroni correction for multiple tests) (Figure 1). These results have three main implications: (i) the majority of proteins are not public goods, i.e. they are intracellular; (ii) the fraction of the genome taken by the secretome is not significantly affected by genome size; (iii) which suggests an important role for secreted proteins in all these prokaryotic clades. Interestingly, this is not the case of other ecologically relevant functions, such as regulatory or sensory proteins, that are highly under-represented in smaller genomes [47].

### Outer-membrane Multi-gene Families are More Abundant

To assess the possible role of intra-chromosomal recombination in the evolution of secreted proteins we identified the multi-gene families in the pan-genomes (see Methods). We found an average of 5.7% multi-genic protein families. These families were unevenly distributed in terms of cell localization (p<0.0001, χ^2^ test on a contingency table) (Table S4). The inner membrane (5.3%) shows the lowest and the outer membrane (8.65%) the highest fraction of multi-gene families. These results suggest that outer membrane proteins are slightly more likely to diversify by intrachromosomal homologous recombination because they are more likely to have homologs in the same genome [48], [49]. This is consistent with the predominance of outer membrane proteins among those subject to variation by homologous recombination [50] and phase variation [51]. To assess how many of these homologs are sufficiently similar to engage in intrachromosomal gene conversion we computed the average protein similarity between orthologs of *E. coli* and *E. fergusonii*. At this genetic distance (average 96.6% protein similarity) the genes are becoming too divergent to engage in homologous recombination [52]. Only a fourth (24.8%) of the multi-gene families in the pan-genomes include pairs of proteins with similarity above this threshold. The cell localization with highest fraction of such multi-gene families is still the outer membrane, but this only concerns 1.9% of all the proteins with this localization. Overall, the fraction of multi-gene families is small suggesting that only a small subset of proteins can evolve rapidly by intra-chromosomal gene conversion.

### The High Genetic Mobility of the Secretome

**Figure 2:**
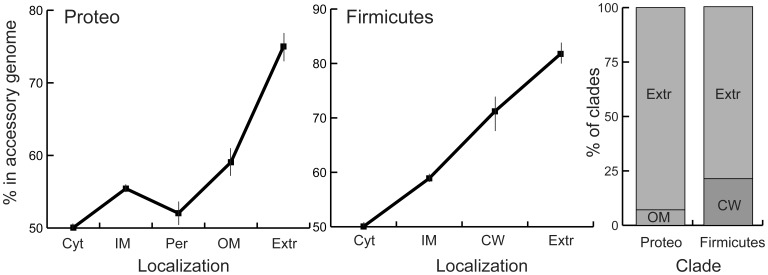
Over-representation of the secretome in the accessory genome. **Left/center.** Percentage of genes in the accessory genome of Proteobacteria/Firmicutes for each category of protein localization in the cell. Vertical bars indicate the limits of the 95% intervals of confidence. The percentage of each localization in the core genome is just 100 minus this value in the accessory genome, e.g. less than 20% of extracellular proteins in Firmicutes are in the core genome. **Right**. Distribution of the protein localizations with highest relative frequency in the accessory genome relative to the core for each clade of Proteobacteria and Firmicutes. Localizations are abbreviated as in Figure 1.

We tested the hypothesis that the secretome is preferably encoded in the accessory genome by first pooling together all clades of Firmicutes and all of Proteobacteria. Indeed, the distributions of protein localizations differed in the core and in the accessory parts of the pan-genomes of both phyla (both p<0.00001, χ^2^ on contingency tables). Genes encoding proteins localized in the cell wall and outer membrane, and especially extracellular proteins, are highly over-represented in the accessory genome relative to the core genome (p<0.0001, χ^2^ test on contingency tables, Table S5). In fact, close to 75% (Proteobacteria) and 82% (Firmicutes) of the genes encoding extracellular proteins are in the accessory genome (p<0.0001, binomial tests) (Figure 2). We then made the same analysis for each clade separately. This confirmed different distributions of protein localizations in terms of accessory and core genomes in 40 out of the 42 clades (p<0.01, same tests). The exceptions were *Methylobacterium* and *Acinetobacter* (resp. p = 0.052 and p = 0.32, same tests). Extracellular proteins were the most over-represented class in the accessory genome in 75% of the clades, followed by outer-membrane (Proteobacteria) and cell wall proteins (Firmicutes) (Figure 2). These results show very clearly that accessory genomes are highly enriched in genes encoding secreted proteins.

The accessory part of pan-genomes is predominantly composed of proteins encoding genes present in a very small number of strains (as low frequency genes) or in a very large number of strains (as high frequency genes) (see graphs and data in Table S3) [53]. Low frequency genes correspond mostly to recently acquired genes whereas high frequency genes correspond mostly to ancestral genes [54]. To test that the secretome is over-represented in the low frequency genes, we divided the accessory genome in two classes: one with genes present in less than half of the genomes (low frequency genes) and the other with the genes present in half or more of the genomes (high frequency genes). As expected, genes encoding secreted proteins were much more frequent among the low frequency genes (Figure S1). Variations in the value separating low and high frequency genes are expected to have little impact in the analysis because gene frequency distributions in pan-genomes are strongly U-shaped [54]. Indeed, the analysis using only strain specific genes and genes present in all but one strain showed similar trends (p<0.0001, Pearson test). Thus the secretome is predominantly encoded in the most mobile part of the accessory genome.

**Figure 3:**
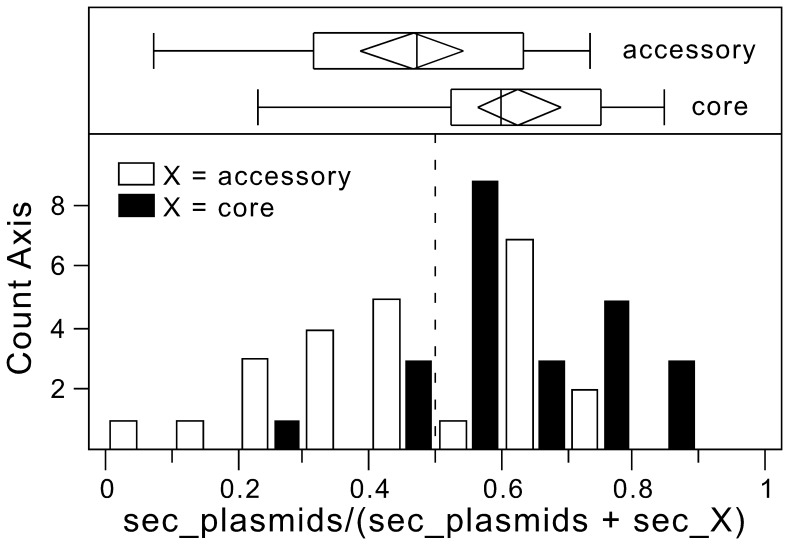
Comparison of the fraction of genes encoding the secretome in plasmids relative to the fraction of genes encoding the secretome in the core and in the accessory genomes. **Lower panel**. We only analyzed clade encoding more than 50 plasmid genes with predicted protein cell localization. For each clade, we computed the fraction of the secretome (extracellular, cell wall, outer membrane) encoded in plasmids (sec_plasmids_) and divided this by the sum of the fraction encoded in the plasmids and the core (black, sec_core_) or the accessory genome (white, sec_accessory_). The precise formulae are sec_plasmids_/(sec_core_+ sec_plasmids_) and sec_plasmids_/(sec_accessory_+ sec_plasmids_). If there are no significant differences between sets then the value should be close to 0.5. Values higher that 0.5 indicate over-representation among plasmids relative to the other set. The graph represents the two histograms. Upper panel. Boxplots of the two distributions represented in the lower panel. Edges of boxplots are the extremes of the distribution, the box represents the 25% and 75% quantiles, the inner line represents the median. Diamonds represent the mean and its 95% interval of confidence.

As mentioned above, we only used chromosomal genes to build the pan-genomes. The exclusion of the few plasmid genes (2.6% of the total) was due to a number of reasons. (i) Cultivation and sequencing procedures often exclude plasmids from complete genomes. For example, all *Shigella flexneri* have the virulence plasmid [55] that is missing in half the genomes in GenBank. Inclusion of plasmids might thus bias the definition of the core genome. (ii) The identification of positional orthologs between plasmids is less reliable than for chromosomes because of their modularity, plasmid fusion/fission and rapid evolution [56]. Reliable identification of positional orthologs is very important to avoid inclusion of hidden paralogs in the analysis of substitution rates. (iii) Plasmids are often in higher copy number than the chromosome [57]. This is associated with inter-replicon recombination and may affect substitution rates. (iv) Plasmids show higher substitution rates than chromosomal genes [58]. This might inflate the substitution rates of elements encoded in plasmids. Mobile elements integrated in the chromosome are much less affected by these problems. Plasmids are expected to over-represent the accessory genome and evolve fast. Furthermore, they were shown in *E. coli* to over-represented secreted proteins [14]. To verify that plasmids genes show trends similar to chromosomal accessory genes we analyzed the 24 clades with more than 50 localized protein families in plasmids. We compared the fraction of the secretome in plasmids, accessory genes and core genes (Figure 3). The frequencies of secreted proteins encoded in plasmids and in the accessory genome were not significantly different (p = 0.4, Wilcoxon signed-rank test), and were higher than in the core genome (both p<0.001, Wilcoxon signed-rank test). Hence, plasmids strongly over-represent genes encoding secreted proteins. Furthermore, the over-representation of genes encoding the secretome in plasmids is statistically indistinguishable from the accessory genome. The exclusion of plasmids from our dataset decreases the size of the accessory genome, but should have no significant effect in the analysis of the genetic mobility of the secretome. We will analyze plasmid data in a subsequent work with a different approach.

### Genes Encoding Secreted Proteins Evolve Faster

**Figure 4:**
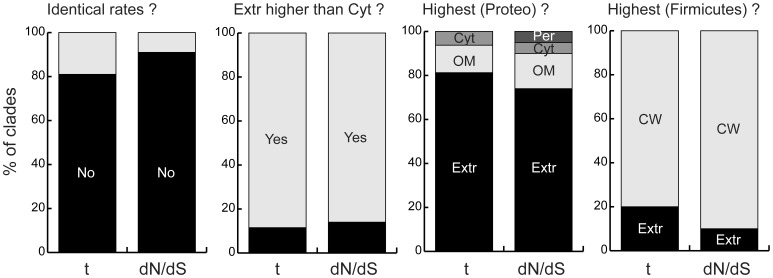
Analysis of substitution rates of genes encoding proteins with different cell localizations. The four graphs correspond to the results of the following analyses (from left to right). (i) The 32 tests, one per clade, that substitution rates are the same for genes encoding proteins with different cell localizations (Wilcoxon test, p<0.05). For the cases where the previous hypothesis was rejected we depict: (ii) the fraction of clades where the average rates in genes encoding extracellular proteins exceed those of genes encoding cytoplasmic proteins, (iii) the protein localization whose genes have highest average values for Proteobacteria and (iv) the same for Firmicutes. In all bars the deviation of the distribution from random is highly significant (p<0.01, binomial or multinomial tests). Localizations are abbreviated as in Figure 1. The labels “t” and “dN/dS” represent the substitution rate per codon and the ratio of non-synonymous over synonymous substitutions.

We studied the association between substitution rates and cell localization to test the hypothesis that genes encoding secreted proteins evolve faster. To avoid the effects of hidden paralogy and gene conversion we excluded from the analysis of substitution rates the few (6%) multi-gene families and the 2.6% of genes encoded in plasmids. Nine clades had too little genetic diversity, i.e. more than half of the genes had zero synonymous (dS) and/or non-synonymous (dN) substitution rates even when comparing the most distant taxa. At this level of divergence the analysis of substitution rates on a per-gene basis is not meaningful and these clades were excluded. One clade had either too closely related genomes or too divergent genomes. It was also rejected to avoid the problem of saturation of synonymous substitutions (see Methods). Hence, we used for this analysis 32 of the 42 clades and a total of 51,193 pairs of orthologs. We computed the synonymous (dS) and non-synonymous (dN) substitution rates, as well as their ratio (dN/dS) and the rate of nucleotide substitutions per codon (t) [59], [60]. We first tested if substitution rates per codon were independent of protein localization (Figure 4). This test was rejected for 16 of the 22 Proteobacteria and for all 10 Firmicutes (p<0.05, but typically p<<0.01, Kruskal-Wallis test, Table S6). Non-synonymous changes have important effects in protein function and their abundance relative to synonymous changes is strong indication of the effect of natural selection in populations [59]. The test of independence of the ratio dN/dS on protein localization was rejected in 19 Proteobacteria and in all 10 Firmicutes (p<0.05, but typically p<<0.01, same test, Table S6). To check whether higher substitution rates in integrated mobile elements affects our conclusions; we made the same analysis using only genes in the core genome. In spite of a smaller sample, especially among secreted proteins that are rare in core genomes, we obtained similar qualitative results. In particular, we found different substitution rates and dN/dS ratios in terms of protein localization in respectively 21 and 24 clades (same tests). This shows that the substitution rates are not independent of cell localization.

In the clades showing statistically significant association between substitution rates and cell localization the extracellular proteins show higher substitution rates than the cytoplasmic proteins (Figure 4). Similar results were observed in the analysis restricted to the core genomes. In this case extracellular proteins were among the Tukey-Kramer HSD [61] class with higher substitution rates (resp. higher dN/dS) in 16 (resp. 19) clades and cell wall or outer membrane proteins in 14 (resp. 16) clades. As a comparison, cytoplasmic proteins were never in the class with highest substitution rate or highest dN/dS. In Proteobacteria, the substitution rates were highest among extracellular proteins. Intriguingly, the majority of Firmicutes showed even higher substitution rates in genes encoding cell wall proteins (both for nucleotide substitution per codon rates and dN/dS, p<0.001, binomial tests, Figure 4). The difference between Firmicutes and Proteobacteria is difficult to interpret because the compartments between phyla are not equivalent. Biophysical constraints acting upon cell wall proteins might be closer to those of periplasmic cell wall associated proteins of Proteobacteria. Unfortunately, current computational methods do not allow identifying the cell wall associated proteins of the periplasm of Proteobacteria to make rigorous comparisons between the two phyla. On the other hand, from an ecological point of view, the cell wall of Firmicutes is analogous to the outer membrane proteins of Proteobacteria in that it is directly exposed to the environment. Outer membrane proteins of Proteobacteria are indeed also evolving faster than the average protein, albeit at a lesser degree than extracellular proteins. These results pinpoint an important difference between Firmicutes and Proteobacteria relating to the rate of evolution of exposed proteins. They also show a critical commonality: in both phyla the secreted proteins show higher substitution rates and accumulate a higher fraction of non-synonymous substitutions, even when they are part of the core genome.

### Effects of the Association between Protein Localization and Virulence Factors

**Figure 5:**
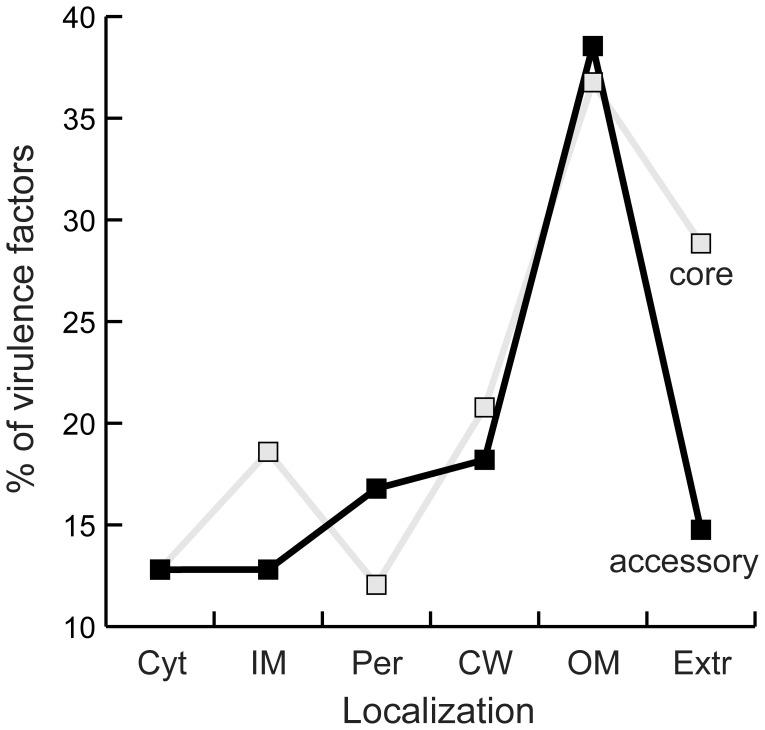
Fraction of homologs of virulence factors in different classes of protein cell localization (see legend of **Figure 1**). The lines correspond to the genes in the core (grey) and in the accessory genome (black).

Many virulence factors are secreted, evolve rapidly and are horizontally transferred [27], [62], [63]. Hence, fast genetic diversification of the secretome could be interpreted as a by-product of the over-representation virulence factors among secreted proteins. To test that secreted virulence factors are not the only cause of the overall rapid evolution of the secretome we used a publicly available database of experimentally verified 2,295 protein virulence factors [64] (see Methods). Homologs of virulence factors are not randomly distributed in terms of protein localization (p<0.0001, χ^2^ test on a contingency table) and are highly over-represented among outer membrane proteins (Figure 5). As a result, we found significant homology to known virulence factors in 25% of the proteins in the secretome and in only 14% of the remaining proteins with predicted cell localization (p<0.0001, same test). Somewhat surprisingly, virulence factors are slightly over-represented in the core genome (7% more than expected, p<0.0001, same test). This small effect might be caused by the presence in our dataset of obligatory pathogens, since the largest over-representation of virulence factors in the core genome is observed in the clades of *Rickettsia* and *Coxiella*. For these bacteria many virulence factors correspond to core functions because pathogenicity is a core trait of the clade. Since virulence factors are not over-represented in the accessory genome, the association between virulence factors and secreted proteins is not causing the over-representation of the secretome in the accessory genome.

Many secreted proteins are known virulence factors, and these are often under positive selection in bacteria [65], [66]. Hence, it could be assumed that high substitution rates in the secretome are caused by the secreted virulence factors. However, on a clade-per-clade analysis we found no evidence that genes encoding secreted virulence factors have higher substitution rates than the rest of the genes encoding secreted proteins. As this could result from lack of statistical power caused by the small number of virulence factors, we pooled together the data on virulence factors and on protein localization from all clades. We made one linear model with t and one other with dN/dS as the dependent variable and as predictors the variables “clade”, “cell localization” and “homology to virulence factors”. The model for the nucleotide substitution rate per codon (t) explained a large fraction of the variance (R^2^ = 0.5663, p<0.0001, F test). Genes encoding virulence factors and genes encoding secreted proteins evolve faster than the other genes (both p<0.0001, t-test). The model for dN/dS explained a smaller fraction of the variance (R^2^ = 0.14813, p<0.0001, F test). Genes encoding virulence factors and genes encoding secreted proteins were associated with higher dN/dS (both p<0.001, t-test). While statistically significant, the effects of removing the variable “homology to virulence factors” from the explanatory variables in the linear models were very small (less than 1% of the R^2^). Furthermore, they were 3 to 30 times smaller than the effects of removing the variable “protein localization”. Hence, the former variable has a much lower explanatory value than the latter. This suggests that the association between secreted proteins and virulence factors is not the major cause of the high substitution rates of the secretome.

Higher substitution rates in secreted proteins relative to virulence factors, could result from the over-representation of the former in the accessory genome (which evolves faster [67]). We therefore made an additional analysis where we compared the substitution rates only of the core genome in terms of protein localization and virulence. We found that results remained qualitatively unchanged when the analysis is restricted to the core genome: we find less than 2% variations in the R^2^ of the linear models when removing the variable “homology to virulence factors”, which has a smaller impact on the R^2^ than the variable “protein localization”. Hence, the gene sequences of virulence factors show higher substitution rates per codon, but the effect of this variable is weaker than the effect of the variable protein localization. Interestingly, while the core genes encoding the secretome exhibit higher than expected dN/dS ratios, this is not the case for the genes encoding virulence factors (median dN/dS = 0.055 versus 0.060 for the rest of the core genome, p<0.0001, Wilcoxon test). This reinforces the previous conclusion that higher rates of substitution per codon and higher dN/dS in the genes encoding the secretome are not trivial consequences of the over-representation of virulence factors in these localizations.

## Discussion

We confirmed the hypothesis that bacterial gene repertoires and sequences encoding secreted proteins evolve fast. Yet, there are a few points that will require further work. Firstly, we excluded the few plasmid genes and thus under-estimated the accessory genomes. In this work we show that plasmid over-represent genes encoding secreted proteins relative to the core genome. Furthermore, the extent of this over-representation is not statistically distinguishable from the one of chromosomal accessory genes. Secondly, we focused our study on proteins and ignored small metabolites. It is easier to identify and study secreted proteins than secondary metabolites, which are sometimes not public goods, just by-products of the cell metabolism. Secreted proteins are more expensive than secondary metabolites and they should thus cause more acute social dilemmas and clearer evolutionary patterns. Yet, since both secreted proteins and secreted metabolites face similar challenges as public goods (social exploitation) and as traits constantly adapting to environmental changes, they are both likely to evolve fast. Thirdly, we showed that genes encoding secreted proteins are over-represented in the accessory genome, evolve fast and show an excess of non-synonymous substitutions, but we have not studied their selection patterns. Notably we have not tried to disentangle in these trends the effects of weaker purifying selection from the effects of positive or diversifying selection. These analyses are very complex at this scale and are ongoing. Fourthly, we have ignored all proteins for which we could not reliably identify cell localization. This left out of the analysis a large fraction of the accessory genome. Nevertheless, substitution rates are higher in the genes encoding secreted proteins even among the core genome, suggesting this should not affect our conclusions.

Within membrane-associated proteins, outer membrane proteins are more frequent in the accessory genome and show higher substitution rates than inner membrane proteins. Within the other localizations, extracellular proteins show the highest frequency in the accessory genome and the highest substitution rates. Thus, association to the membrane, which affects protein evolution [68], is not enough to explain the observed rapid evolution of the secretome. Secreted proteins are thought to endure strong selection for lower cost amino acids [14], [69]. This effect should slow down, not accelerate, their substitution rates. Recently acquired genes tend to be shorter, of unknown function, have atypical sequence composition, lack homologs in the databanks and evolve faster [67], [70], [71]. To control for this effect, we showed higher substitution rates also among core-genome encoded secreted proteins. Extracellular and exposed proteins have key roles in ecological interactions of bacteria and are over-represented in studies of positive and diversifying selection [23], [25], [26]. Hence, the high substitution rates of the secretome and the over-representation of these genes in the accessory genome are likely to be adaptive.

Our work shows that virulence factors do not diversify as fast as other secreted proteins: they have lower substitution rates, lower dN/dS and are less frequent in the accessory genome. Naturally, our conclusions are dependent on the quality of the data from VFDB, which previous works indicate as the most accurate available databank on virulence factors [63]. Importantly, our results show rapidly evolving secretomes in bacteria that are not pathogenic, notably the mutualists *Rhizobium* and *Cupriavidus tawanensis*, and the free-living *Geobacillus* and *Shewanella*. Hence, rapid evolution of the secretome does not depend strictly on pathogenicity. Mechanisms of pathogenesis have naturally been subject to extensive scrutiny by the scientific community, but in many respects they are like many other processes that allow bacteria to scavenge their environment for nutrients [72]. Interestingly, among virulence factors, the effectors of type 3 and type 4 secretion systems are over-represented in mobile elements and genomic islands [28], [63], [73]. Hence, secreted virulence factors, but not necessarily all virulence factors, are genetically mobile. This is a trait they share with the remaining secreted proteins, even the ones not involved in virulence. The interactions of bacteria with multicellular eukaryotes, grazing protozoa and phages lead to very fast molecular recognition arms races that are expected to lead to positive and/or balancing selection in exposed and in extracellular proteins [74], [75]. Social dilemmas caused by the cooperative production of public goods are also expected to lead to rapid evolution of the secretome [14]. Our results are in agreement with the hypothesis that these different factors lead to the rapid evolution of secreted proteins.

## Methods

### Data on Genomes, Virulence Factors and Protein Localization

Complete genome sequences and their annotations were retrieved from GenBank RefSeq (ftp://ftp.ncbi.nih.gov/). We excluded pseudogenes. The 16S rDNA sequences were retrieved from the genomes using their annotations and their precise limits manually corrected when needed. Data on 2295 virulence factors was retrieved from VFDB [64]. Genes homologous to virulence factors were identified using Fasta (v36, [76]) selecting for hits with an e-value<10^−5^, and more than 50% protein similarity along at least 70% of the size of the smallest protein. We discarded hits when one of the proteins was less than half the size of the other. A more permissive definition of homology (only e-value <10^−5^) provided qualitatively similar results (data not shown). Protein localization of the representative of each protein family was computed with PsortB 3.1 [45], using the gram-positive predictor for Firmicutes and the gram-negative predictor for Proteobacteria. We discarded the proteins without a reliable prediction score and the very few proteins with predicted multiple locations. The secretome is defined as the set of outer membrane and extracellular proteins in Proteobacteria and extracellular and cell wall proteins in Firmicutes.

### Definition of Core and Pan Genomes

A preliminary set of orthologs was defined by identifying unique pairwise reciprocal best hits, with at least 60% similarity in amino acid sequence and less than 20% of difference in protein length. We refined this list using the distribution of similarity of these putative orthologs and gene order conservation (as in [77]). The analysis of orthology was made for every pair of genomes in each clade. The primary core genome of a clade was defined as the intersection of the pairwise lists of positional orthologs and was used to build the phylogenetic tree (see below). The pan-genome was defined as the repertoire of gene families found in at least one strain of a given clade. We merged together in a single family of the pan-genomes the families of positional orthologs sharing more than 60% similarity and less than 20% of difference in length. Hence, the pan-genome families can have more than one gene per genome, which allows putting together very closely related homologs that in general have similar functions and cell localizations. The final core gene set was defined from the pan-genomes as the set of gene families with at least one representative per genome. The sizes and compositions of the primary and final core genomes are very similar (R^2^ = 0.99, p<0.0001).

### Phylogenetic Analysis

We computed a multiple alignment for each protein family of the core genome using muscle v3.6 (default parameters) [78] and back-translated alignments to DNA. The orthologous proteins encoded by closely related genomes are typically more than 95% identical and there is no need to manually correct these alignments. The distance matrix between taxa was computed from the concatenated alignments of the core genome with Tree-puzzle 5.2 [79] (ML model HKY+Γ(8)+I). The tree of the core genome was built from the distance matrix using BioNJ [80]. All trees were visually inspected. Taxa showing large genetic distances with the main group of genomes were excluded (terminal branches with >0.1 subst/nt).

### Analysis of Substitution Rates

Substitution rates between pairs of orthologs were computed using PAML 4 [81]. Since the sample size is very large and we were only interested in pairwise rates we used the method implemented in the program yn00 with default parameters [60]. We then analyzed in each clade the distributions of synonymous (dS), non-synonymous substitutions (dN), their ratio (dN/dS) and the number of nucleotide substitutions per codon (t). Rates were computed for genes between a pair of taxa within the clade. The taxa were chosen such that their genetic distances were sufficiently large (median dS and median dN per gene higher than zero), but below saturation (less than 5% of genes with t>1.5). The 10 clades lacking such an appropriate pair of taxa were discarded. To avoid introducing closely related paralogs in our analysis, we excluded the 6% protein families with multiple copies of a gene in any genome of the clade.

## Supporting Information

Figure S1 **Observed/expected ratio of the proteins per localization category among the three classes of core genome, accessory and present in more than 50% of the genomes (high frequency genes) and accessory present in less than 50% of the genomes (low frequency genes).** Proteobacteria data on the left and Firmicutes data on the right.
(TIF)

Table S1 **Description of clades from Proteobacteria used in the study.** The table displays the number of genomes per clade, the average number of proteins per clade, the pangenome size and its decomposition in core and accessory genes, the number of proteins with predicted cell localization, the number of multigenic families, i.e. families with more than one member in any given genome, and the number of homologs to virulence factors.
(DOC)

Table S2 **Description of clades from Proteobacteria used in the study.** The table displays the number of genomes per clade, the average number of proteins per clade, the pangenome size and its decomposition in core and accessory genes, the number of proteins with predicted cell localization, the number of multigenic families and the number of homologs to virulence factors.
(DOC)

Table S3 **Genomes used in the study, classification in clades, pan-genome and its spectrum of frequencies.**
(XLS)

Table S4 **Contingency table of cell localization by multi-gene families.** First line in cell is the count, second line is the percentage relative to the cell localization column and the third line is the expected value. Abbreviations of cell localization: cytoplasm (Cyt), inner membrane (IM), periplasm (Per, Proteobacteria), cell wall (CW, Firmicutes), outer membrane (OM, Proteobacteria) and extracellular (Extr).
(DOC)

Table S5 **Tests regarding genetic repertoires of the pan-genome.** Columns depict: (ii) p-value of the test of independence of protein localization, (iii) localization with the lowest fraction of genes in the core (relative to the non-core genome), (iv & v) ratio of genes in core/accessory for extracellular proteins and for outer-membrane (cell wall in Firmicutes).
(DOC)

Table S6 **Summary of tests for the substitution rates of the pan-genome.**
(DOC)
