# Supplementary figures and images for: Rapid Evolution of the Sequences and Gene Repertoires of Secreted Proteins in Bacteria
Source: PLoS One. 2012 Nov 26;7(11):e49403. doi: 10.1371/journal.pone.0049403 (PMC3506625; doi:10.1371/journal.pone.0049403)

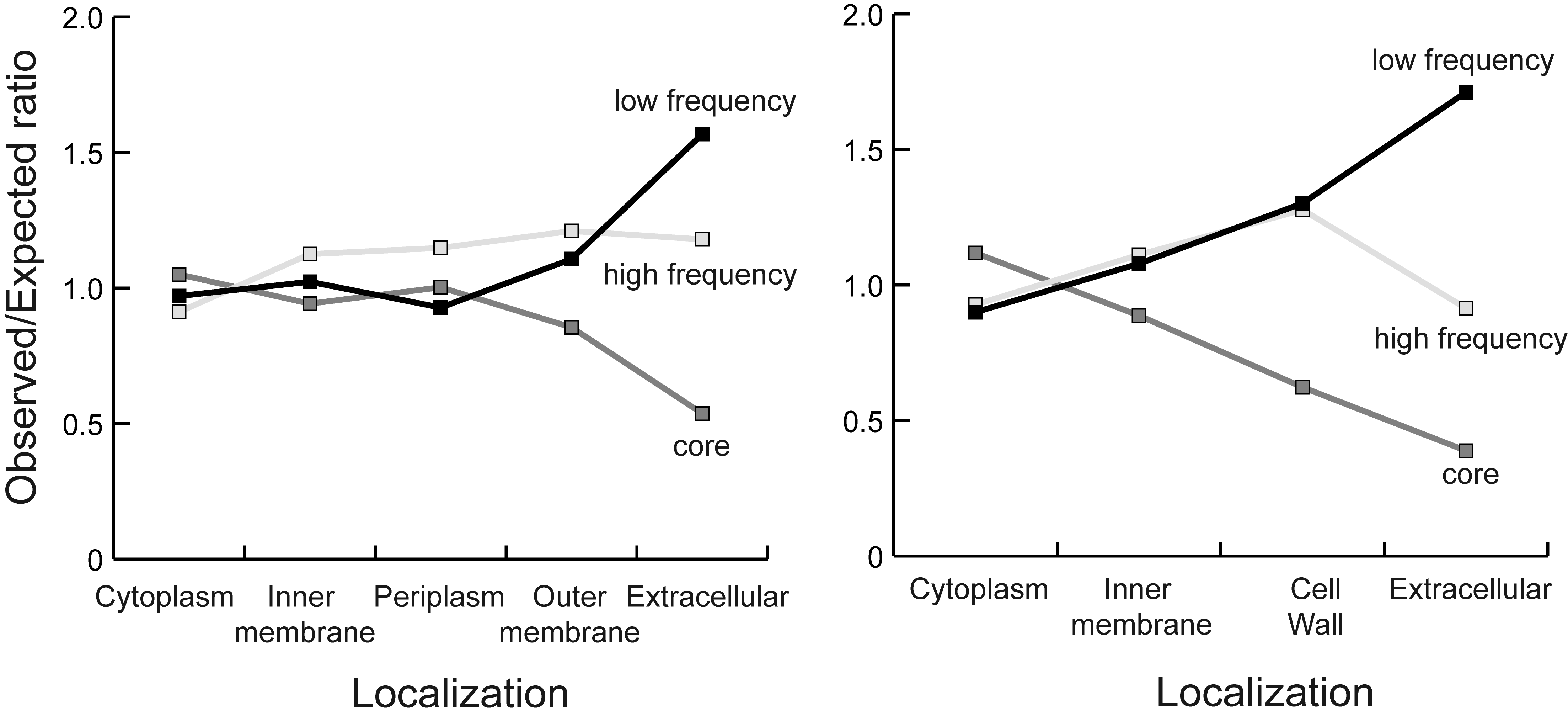

Supplement: Figure S1 — Observed/expected ratio of the proteins per localization category among the three classes of core genome, accessory and present in more than 50% of the genomes (high frequency genes) and accessory present in less than 50% of the genomes (low frequency genes). Proteobacteria data on the left and Firmicutes data on the right. (TIF) [file pone.0049403.s001.tif]
