# Supplementary material for: Rapid Evolution of the Sequences and Gene Repertoires of Secreted Proteins in Bacteria
Source: PLoS One. 2012 Nov 26;7(11):e49403. doi: 10.1371/journal.pone.0049403 (PMC3506625; doi:10.1371/journal.pone.0049403)
Supplement: Table S1 — Description of clades from Proteobacteria used in the study. The table displays the number of genomes per clade, the average number of proteins per clade, the pangenome size and its decomposition in core and accessory genes, the number of proteins with predicted cell localization, the number of multigenic families, i.e. families with more than one member in any given genome, and the number of homologs to virulence factors. (DOC) [file pone.0049403.s002.doc]

| **Clades** | *• Species name* | | | | | | | | | |
| --- | --- | --- | --- | --- | --- | --- | --- | --- | --- | --- |
|  | **No. of Genomes** | **Average no. of genes** | **Pangenome** | | **No. of predicted localization** | **No. of Multigenic families** | **No. VF** |  |  | |
|  |  |  | **Core Genes** | **Accessory Genes** |  |  |  |  |  |  |
|  | | | | | | | | | | |
| acba | *•Acinetobacter baumannii* | | | | | | | | | |
|  | 8 | 3578 | 1275 | 5298 | 4268 | 2305 | 685 |  | |  |
| bagr | *•Bartonella grahami •B. henselae •B. quintana •B. tribocorum* | | | | | | | | | |
|  | 4 | 1610 | 1043 | 808 | 1232 | 619 | 183 |  | |  |
| brab | *•Brucella abortus •B. ovis •B. canis •B. melitensis •B. microti •B. pinnipedialis •B. suis* | | | | | | | | | |
|  | 13 | 3185 | 1671 | 3425 | 2968 | 55 | 457 |  | |  |
| brja | *•Bradyrhizobium japonicum •Rhodopseudomonas palustris* | | | | | | | | | |
|  | 6 | 6233 | 2362 | 11919 | 8620 | 977 | 1445 |  | |  |
| caje | *•Campylobacter jejuni* | | | | | | | | | |
|  | 9 | 1658 | 976 | 1927 | 1994 | 90 | 269 |  | |  |
| cobu | *•Coxiella burnetii* | | | | | | | | | |
|  | 5 | 1903 | 1290 | 1295 | 1576 | 37 | 211 |  | |  |
| cune | *•Cupriavidus necator •C. taiwanensis •Ralstonia eutropha* | | | | | | | | | |
|  | 4 | 5872 | 3281 | 6184 | 6179 | 513 | 1384 |  | |  |
| eram | *•Erwinia amylovora •E. pyrifoliae •E. tasmaniensis* | | | | | | | | | |
|  | 6 | 3618 | 2394 | 3561 | 3214 | 252 | 748 |  | |  |
| esco | *•Escherichia coli* | | | | | | | | | |
|  | 47 | 4658 | 1989 | 11721 | 6803 | 1262 | 1443 |  | |  |
| frtu | •*Francisella tularensis* | | | | | | | | | |
|  | 9 | 1599 | 1087 | 1297 | 1655 | 63 | 246 |  | |  |
| hain | *•Haemophilus influenzae* | | | | | | | | | |
|  | 9 | 1728 | 1009 | 1899 | 1957 | 103 | 287 |  | |  |
| hepy | *•Helicobacter pylori* | | | | | | | | | |
|  | 30 | 1533 | 957 | 3168 | 2037 | 131 | 231 |  | |  |
| klpn | *•Klebsiella pneumoniae •K. variicola* | | | | | | | | | |
|  | 4 | 5060 | 3736 | 2617 | 4346 | 347 | 937 |  | |  |
| lepn | *•Legionella pneumophila* | | | | | | | | | |
|  | 5 | 3048 | 2277 | 1741 | 2660 | 181 | 366 |  | |  |
| mech | *•Methylobacterium chloromethanicum •M. extorquens* | | | | | | | | | |
|  | 4 | 5139 | 3631 | 3290 | 3853 | 515 | 562 |  | |  |
| neme | *•Neisseria meningitidis* | | | | | | | | | |
|  | 14 | 1950 | 1261 | 1886 | 1734 | 196 | 228 |  | |  |
| psae | *•Pseudomonas aeruginosa* | | | | | | | | | |
|  | 5 | 5870 | 4458 | 2890 | 4892 | 419 | 952 |  | |  |
| psen | *•Pseudomonas entomophila •P. putida* | | | | | | | | | |
|  | 7 | 5214 | 3091 | 6131 | 6126 | 619 | 1368 |  | |  |
| raso | *•Ralstonia solanacearum* | | | | | | | | | |
|  | 5 | 3332 | 2205 | 3151 | 3051 | 214 | 549 |  | |  |
| rhet | *•Rhizobium etli •R. leguminosarum* | | | | | | | | | |
|  | 5 | 4391 | 2918 | 3790 | 4262 | 251 | 734 |  | |  |
| rhpa | *•Rhodopseudomonas palustris* | | | | | | | | | |
|  | 4 | 4711 | 2564 | 5207 | 4945 | 412 | 890 |  | |  |
| riaf | *•Rickettsia africae •R. akari •R. canadensis •Ri. conorii •R. felis •R. heilongjiangensis •R. massiliae •R. peacockii •R. prowazekii •R. rickettsi •R. typhi* | | | | | | | | | |
|  | 13 | 1130 | 641 | 2983 | 1384 | 43 | 114 |  | |  |
| saen | *•Salmonella enterica subsp. enterica* | | | | | | | | | |
|  | 20 | 4520 | 2600 | 5876 | 4347 | 432 | 869 |  | |  |
| shba | *•Shewanella baltica •S. oneidensis •S. putrefaciens* | | | | | | | | | |
|  | 13 | 4198 | 2622 | 5712 | 5053 | 425 | 987 |  | |  |
| simd | *•Sinorhizobium medicae •S. meliloti* | | | | | | | | | |
|  | 4 | 3478 | 2731 | 1579 | 2843 | 147 | 484 |  | |  |
| vich | *•Vibrio cholerae* | | | | | | | | | |
|  | 6 | 3708 | 2731 | 1855 | 3063 | 206 | 536 |  | |  |
| xyfa | *•Xylella fastidiosa* | | | | | | | | | |
|  | 5 | 2249 | 1496 | 1835 | 1669 | 191 | 236 |  | |  |
| yepe | *•Yersinia pestis •Y. pseudotuberculosis* | | | | | | | | | |
|  | 15 | 3936 | 2219 | 4413 | 3882 | 239 | 817 |  | |  |
| Total | 280 | 99109 | 60515 | 107458 | 100613 (60%) | 9005 (5%) | 18218 (11%) |  | |  |
