# Supplementary material for: Rapid Evolution of the Sequences and Gene Repertoires of Secreted Proteins in Bacteria
Source: PLoS One. 2012 Nov 26;7(11):e49403. doi: 10.1371/journal.pone.0049403 (PMC3506625; doi:10.1371/journal.pone.0049403)
Supplement: Table S2 — Description of clades from Proteobacteria used in the study. The table displays the number of genomes per clade, the average number of proteins per clade, the pangenome size and its decomposition in core and accessory genes, the number of proteins with predicted cell localization, the number of multigenic families and the number of homologs to virulence factors. (DOC) [file pone.0049403.s003.doc]

| **Clades** | *• Species name* | | | | | | | | | | |
| --- | --- | --- | --- | --- | --- | --- | --- | --- | --- | --- | --- |
|  | **No. of Genomes** | **Average no. of genes** | **Pan Genes** | | **No. of predicted localization** | **No. of Multigenic families** | **No. VF** |  | |  | |
|  |  |  | **Core Genes** | **Accessory Genes** |  |  |  |  |  |  |  |
| **Firmicutes (monoderms)** | | | | | | | | | | | |
| baam | *•Bacillus amyloliquefaciens* | | | | | | | | | | |
|  | 4 | 3973 | 2979 | 1987 | 3653 | 205 | 504 | |  | |  |
| baan | *•Bacillus anthracis •B. cereus •B. thuringiensis •B. weihenstephanensis* | | | | | | | | | | |
|  | 21 | 5290 | 2488 | 9650 | 8420 | 795 | 919 | |  | |  |
| clbo | *•Clostridium botulinum* | | | | | | | | | | |
|  | 10 | 3629 | 2212 | 3695 | 4107 | 358 | 555 | |  | |  |
| geka | *•Geobacillus kaustophilus* | | | | | | | | | | |
|  | 4 | 3409 | 2558 | 1554 | 3467 | 175 | 455 | |  | |  |
| laca | *•Lactobacillus casei* | | | | | | | | | | |
|  | 5 | 2971 | 1985 | 1688 | 2584 | 192 | 319 | |  | |  |
| lade | *•Lactobacillus delbrueckii* | | | | | | | | | | |
|  | 4 | 1771 | 1109 | 1589 | 1922 | 146 | 234 | |  | |  |
| lala | *•Lactococcus lactis* | | | | | | | | | | |
|  | 6 | 2398 | 1418 | 2367 | 2733 | 260 | 328 | |  | |  |
| limo | *•Listeria monocytogenes* | | | | | | | | | | |
|  | 12 | 2942 | 2279 | 1506 | 2915 | 165 |  | |  | |  |
| stau | *•Staphylococcus aureus* | | | | | | | | | | |
|  | 24 | 2641 | 1666 | 2875 | 3150 | 363 | 383 | |  | |  |
| steq | •*Streptococcus equi* | | | | | | | | | | |
|  | 4 | 1961 | 1359 | 1253 | 1958 | 122 | 266 | |  | |  |
| stmi | *•Streptococcus mitis •S. pneumoniae* | | | | | | | | | | |
|  | 19 | 2084 | 1074 | 3729 | 3390 | 254 | 380 | |  | |  |
| stpy | *•Streptococcus pyogenes* | | | | | | | | | | |
|  | 13 | 1853 | 1134 | 2049 | 2259 | 157 | 272 | |  | |  |
| stsa | *•Streptococcus salivarius •S. thermophilus* | | | | | | | | | | |
|  | 7 | 1911 | 1099 | 2496 | 2605 | 1911 | 343 | |  | |  |
| stsu | *•Streptococcus suis* | | | | | | | | | | |
|  | 8 | 2002 | 1198 | 2127 | 2524 | 106 | 291 | |  | |  |
| Total | 141 | 38835 | 24558 | 38565 | 45687 (72%) | 3422 (5%) | 5638(9%) | |  | |  |
