# Supplementary material for: Rapid Evolution of the Sequences and Gene Repertoires of Secreted Proteins in Bacteria
Source: PLoS One. 2012 Nov 26;7(11):e49403. doi: 10.1371/journal.pone.0049403 (PMC3506625; doi:10.1371/journal.pone.0049403)
Supplement: Table S4 — Contingency table of cell localization by multi-gene families. First line in cell is the count, second line is the percentage relative to the cell localization column and the third line is the expected value. Abbreviations of cell localization: cytoplasm (Cyt), inner membrane (IM), periplasm (Per, Proteobacteria), cell wall (CW, Firmicutes), outer membrane (OM, Proteobacteria) and extracellular (Extr). (DOC) [file pone.0049403.s005.doc]

| Count  Col %  Expected | CW | Cyt | IM | Extr | OM | Per | Total |
| --- | --- | --- | --- | --- | --- | --- | --- |
| Multi-gene | 54  6.94  44.07 | 4969  5.68  4957.36 | 2543  5.26  2740.09 | 231  6.77  193.26 | 222  8.65  145.29 | 268  7.34  206.92 | 8287 |
| Others | 724  93.06  733.93 | 82549  94.32  82560.6 | 45831  94.74  45633.9 | 3181  93.23  3218.73 | 2343  91.35  2419.71 | 3385  92.66  3446.08 | 138013 |
| Total | 778 | 87518 | 48374 | 3412 | 2565 | 3653 | 146300 |
