# Supplementary material for: Rapid Evolution of the Sequences and Gene Repertoires of Secreted Proteins in Bacteria
Source: PLoS One. 2012 Nov 26;7(11):e49403. doi: 10.1371/journal.pone.0049403 (PMC3506625; doi:10.1371/journal.pone.0049403)
Supplement: Table S5 — Tests regarding genetic repertoires of the pan-genome. Columns depict: (ii) p-value of the test of independence of protein localization, (iii) localization with the lowest fraction of genes in the core (relative to the non-core genome), (iv & v) ratio of genes in core/accessory for extracellular proteins and for outer-membrane (cell wall in Firmicutes). (DOC) [file pone.0049403.s006.doc]

| clades | p-value | rarest in core | extracellular | OM/CW | phylum |
| --- | --- | --- | --- | --- | --- |
| acba | 0.3173 | extrac | 0.161 | 0.333 | diderm |
| baam | 0.0001 | extrac | 0.705 | 4.800 | monoderm |
| baan | 0.0001 | extrac | 0.101 | 0.229 | monoderm |
| bagr | 0.0001 | extrac | 0.417 | 0.714 | diderm |
| brab | 0.0001 | extrac | 0.556 | 0.600 | diderm |
| brja | 0.0001 | extrac | 0.070 | 0.234 | diderm |
| caje | 0.0069 | OM | 0.556 | 0.515 | diderm |
| clbo | 0.0001 | extrac | 0.221 | 1.000 | monoderm |
| cobu | 0.0001 | extrac | 1.000 | 2.167 | diderm |
| cune | 0.002 | extrac | 0.422 | 0.670 | diderm |
| eram | 0.0001 | extrac | 0.530 | 1.250 | diderm |
| esco | 0.0001 | extrac | 0.037 | 0.219 | diderm |
| frtu | 0.0001 | extrac | 0.500 | 1.700 | diderm |
| geka | 0.0001 | extrac | 0.871 | 1.636 | monoderm |
| hain | 0.0016 | extrac | 0.143 | 0.500 | diderm |
| hepy | 0.0001 | extrac | 0.432 | 0.913 | diderm |
| klpn | 0.0007 | extrac | 0.800 | 2.960 | diderm |
| laca | 0.0001 | extrac | 0.114 | 0.607 | monoderm |
| lade | 0.0001 | extrac | 0.186 | 0.320 | monoderm |
| lala | 0.0001 | extrac | 0.140 | 0.167 | monoderm |
| lepn | 0.001 | extrac | 1.650 | 4.500 | diderm |
| limo | 0.0001 | extrac | 0.605 | 0.744 | monoderm |
| mech | 0.0517 | extrac | 1.000 | 2.190 | diderm |
| neme | 0.0001 | extrac | 0.222 | 0.658 | diderm |
| psae | 0.0001 | extrac | 0.735 | 1.892 | diderm |
| psen | 0.0001 | extrac | 0.181 | 0.439 | diderm |
| raso | 0.0006 | extrac | 0.453 | 1.132 | diderm |
| rhet | 0.0001 | extrac | 0.372 | 2.100 | diderm |
| rhpa | 0.0001 | extrac | 0.186 | 0.411 | diderm |
| riaf | 0.0001 | extrac | 0.154 | 0.364 | diderm |
| saen | 0.0001 | extrac | 0.233 | 0.677 | diderm |
| shon | 0.0001 | extrac | 0.242 | 0.353 | diderm |
| simd | 0.0001 | extrac | 0.500 | 1.438 | diderm |
| stau | 0.0001 | extrac | 0.188 | 0.447 | monoderm |
| steq | 0.0001 | extrac | 0.708 | 1.115 | monoderm |
| stmi | 0.0001 | extrac | 0.081 | 0.117 | monoderm |
| stpy | 0.0001 | CW | 0.245 | 0.213 | monoderm |
| stsa | 0.0001 | CW | 0.113 | 0.033 | monoderm |
| stsu | 0.0001 | CW | 0.250 | 0.239 | monoderm |
| vich | 0.0132 | extrac | 1.238 | 3.167 | diderm |
| xyfa | 0.0001 | extrac | 0.733 | 2.429 | diderm |
| yeps | 0.0001 | OM | 0.484 | 0.410 | diderm |
