# Supplementary material for: Rapid Evolution of the Sequences and Gene Repertoires of Secreted Proteins in Bacteria
Source: PLoS One. 2012 Nov 26;7(11):e49403. doi: 10.1371/journal.pone.0049403 (PMC3506625; doi:10.1371/journal.pone.0049403)
Supplement: Table S6 — Summary of tests for the substitution rates of the pan-genome. (DOC) [file pone.0049403.s007.doc]

|  | t |  |  |  | dN/dS |  |  |  |  |
| --- | --- | --- | --- | --- | --- | --- | --- | --- | --- |
| Clade | kruskal test of homogeneity | |  |  | kruskal test of homogeneity | |  |  |  |
|  | p-value | result | extr > cyt? | top location | p-value | result | extrac > cyt? | top location | class |
| acba | 0.0372 | Reject | Yes | extrac | 0.0269 | Reject | No | OM | diderm |
| baam | 0.0001 | Reject | Yes | CW | 0.0001 | Reject | Yes | CW | monoderm |
| baan | 0.0001 | Reject | Yes | CW | 0.0001 | Reject | Yes | CW | monoderm |
| bagr | 0.0003 | Reject | No | OM | 0.0001 | Reject | No | OM | diderm |
| caje | 0.0179 | Reject | Yes | extrac | 0.0996 | Not reject | Yes | OM | diderm |
| clbo | 0.0001 | Reject | Yes | CW | 0.0001 | Reject | Yes | extrac | monoderm |
| cune | 0.0001 | Reject | Yes | extrac | 0.0001 | Reject | Yes | extrac | diderm |
| eram | 0.0003 | Reject | Yes | extrac | 0.0001 | Reject | Yes | extrac | diderm |
| esco | 0.2000 | Not reject | Yes | extrac | 0.0117 | Reject | Yes | extrac | diderm |
| frtu | 0.1300 | Not reject | Yes | extrac | 0.009 | Reject | Yes | peripl | diderm |
| geka | 0.0001 | Reject | Yes | CW | 0.0001 | Reject | Yes | CW | monoderm |
| hepy | 0.0077 | Reject | Yes | extrac | 0.0006 | Reject | No | cyt | diderm |
| klpn | 0.0112 | Reject | Yes | extrac | 0.0023 | Reject | Yes | extrac | diderm |
| laca | 0.0001 | Reject | No | CW | 0.0001 | Reject | No | CW | monoderm |
| lade | 0.0001 | Reject | Yes | extrac | 0.0001 | Reject | Yes | CW | monoderm |
| lepn | 0.387 | Not reject | Yes | OM | 0.1 | Not reject | No | cyt | diderm |
| limo | 0.0125 | Reject | Yes | extrac | 0.0001 | Reject | Yes | CW | monoderm |
| mech | 0.312 | Not reject | No | peripl | 0.0088 | Reject | Yes | extrac | diderm |
| neme | 0.0017 | Reject | No | cyt | 0.0001 | Reject | Yes | extrac | diderm |
| psae | 0.074 | Not reject | Yes | extrac | 0.0001 | Reject | Yes | OM | diderm |
| psen | 0.0001 | Reject | Yes | extrac | 0.0001 | Reject | Yes | extrac | diderm |
| raso | 0.0001 | Reject | Yes | extrac | 0.0001 | Reject | Yes | extrac | diderm |
| rhet | 0.0248 | Reject | Yes | extrac | 0.0001 | Reject | Yes | extrac | diderm |
| riaf | 0.024 | Reject | Yes | OM | 0.011 | Reject | Yes | extrac | diderm |
| saen | 0.0006 | Reject | Yes | extrac | 0.0001 | Reject | Yes | extrac | diderm |
| shon | 0.0001 | Reject | Yes | extrac | 0.0001 | Reject | Yes | extrac | diderm |
| simd | 0.0377 | Reject | Yes | extrac | 0.9 | Not reject | No | peripl | diderm |
| stau | 0.0001 | Reject | Yes | CW | 0.0011 | Reject | Yes | CW | monoderm |
| steq | 0.0001 | Reject | Yes | CW | 0.0001 | Reject | Yes | CW | monoderm |
| stmi | 0.0001 | Reject | Yes | CW | 0.0001 | Reject | Yes | CW | monoderm |
| vich | 0.69 | Not reject | Yes | IM | 0.0028 | Reject | Yes | extrac | diderm |
| yeps | 0.0001 | Reject | Yes | extrac | 0.0096 | Reject | Yes | extrac | diderm |
